# Ultrastructure and morphology of antennal sensilla of the adult diving beetle *Cybister japonicus* Sharp

**DOI:** 10.1371/journal.pone.0174643

**Published:** 2017-03-30

**Authors:** Li-Mei Song, Xue-Min Wang, Jian-Ping Huang, Fang Zhu, Xiang Jiang, Shan-Gan Zhang, Li-Ping Ban

**Affiliations:** 1 Department of Grassland Science, College of Animal Science and Technology, China Agricultural University, Beijing, China; 2 Institute of Animal Science, Chinese Academy of Agricultural Sciences, Beijing, China; 3 Department of Entomology, Washington State University, Pullman, Washington, United States of America; 4 Huangpu Entry-Exit Inspection and Quarantine Bureau, Guangdong, China; 5 State Key Laboratory of Integrated Management of Pest Insects and Rodents, Institute of Zoology, Chinese Academy of Sciences, Beijing, China; University of Arizona, UNITED STATES

## Abstract

The morphology and distribution of the antennal sensilla of adult diving beetle *Cybister japonicus* Sharp (Dytiscidae, Coleoptera), have been examined. Five types of sensilla on the antennae were identified by scanning electron microscope (SEM) and transmission electron microscope (TEM). Sensilla placodea and elongated s. placodea are the most abundant types of sensilla, distributing only on the flagellum. Both these types of sensilla carry multiple pore systems with a typical function as chemoreceptors. Three types of s. coeloconica (Type I–III) were also identified, with the characterization of the pit-in-pit style, and carrying pegs externally different from each other. Our data indicated that both type I and type II of s. coleconica contain two bipolar neurons, while the type III of s. coleconica contains three dendrites in the peg. Two sensory dendrites in the former two sensilla are tightly embedded inside the dendrite sheath, with no space left for sensilla lymph. There are no specific morphological differences in the antennal sensilla observed between males and females, except that the males have longer antennae and more sensilla than the females.

## Introduction

Chemoreceptor structures, e.g. insect antennae, play a very important role in many crucial insect behaviors, such as searching for food, mating, localization for oviposition, aggregation, and escaping from dangers [1–3]. These structures are especially important for the aquatic insects which live in the turbid, dark, highly complex habitat conditions or species with poor vision capabilities [4, 5]. In insects, external chemical signals are mainly detected by specialized sensilla located on the chemoreceptor structures [6–9]. Insect antennal sensilla which usually occur in form of hairs, pegs, and pits can be termed as sensilla trichodea, placodea, coeloconica, basiconica, chaetica, Bӧhm’s bristles, etc. based on their morphological characteristics [10, 11]. These sensilla can also be classified into multiporous, uniporous and aporous sensilla according to their structural features. Generally, multiporous and uniporous sensilla function as chemoreceptors (e.g. olfactory and gustatory), while aporous sensilla function as mechanoreceptors, thermoreceptors, hygroreceptors, or CO_2_ receptors [11–14].

Although it is quite different from terrestrial environments, the aquatic environment is well suit to solute and disperse chemical information [5]. Aquatic insects have evolved highly sensitive receptors for the chemical information detection, which help in foraging, mating, reproduction, and assessment of predation risk [15]. For example, Schaller (1926) [16] reported that *Dytiscus marginalis* has very well developed chemical receptors located on antennae (odor) or maxillary and labial palpi (taste), which are especially critical for detecting potential food.

The diving beetle *Cybister japonicus* Sharp (Coleoptera: Dytiscidae) lives in stagnant waters [17, 18] during both larval and adult stages and represents an important aquatic predator. The first- and second-instar larvae of *C*. *japonicus* prey mainly on insects, whereas the third-instar larvae fed on both insects and vertebrates [19]. Adult diving beetles could be the efficient predators of mosquito larvae [20].

The Dytiscidae (diving beetle) are among the most common insect inhabitants of freshwaters, however, morphology and ultrastructural studies on the sensilla are still scant. Thus far, only few species of diving beetle had been studied, including *Graphoderus occidentalis* [21, 22], *Acilius sulcatus* [23], and *Laccophilus maculosus* [24].

To the best of our knowledge, the structure of antennal sensilla in *C*. *japonicus* Sharp has not been documented in detail so far. Here, we examined the type, number, distribution, external morphology and internal structures of antennal sensilla of the diving beetle *C*. *japonicus* Sharp using both scanning electron microscope (SEM) and transmission electron microscope (TEM). The information generated here might reveal possible structure-function relationships and improve our understanding in physiological and behavioral functions of antennal sensilla in this species.

## Materials and methods

### Insect collections

The adult diving beetle *Cybister japonicus* Sharp used in this study was obtained from the Huangsha Aquatic Products Trading Market in Guangzhou, Guangdong Province, China (23°06′N, 113°14′E), and reared in the lab at room temperature (15–20°C) in water, with a photoperiod of 18L: 6D. All adults, around 100 beetles in total, were kept in the 0.5m*0.4m*0.4m aquariums filled with tap water and covered with fly screen to prevent beetles from flying.

### Light microscope (LM) observation

Antennae were treated with 10% sodium hydroxide overnight and dehydrated by immersion in 100% ethanol, followed by 1:1 ethanol/xylene, and 100% xylene. The antennae were spread on a slide and mounted in Canadian gum. Ten antennae were used for both males and females.

### Scanning electron microscope (SEM) observation

Ten insects for each gender were used for SEM observations. Antennae were dissected from the head and fixed in 70% ethanol for 2 hrs, then cleaned in ultrasonic bath (KQ118, Kunshan, China) for 1 min in the same solution, and then dehydrated in a series of graded solution of ethanol, from 70% to 100%. After treatment with 100% ethanol for 30 min, the samples were critical-point dried with a critical point dryer (LEICA CPD 030, Wetzlar, Germany). The dried antennae were mounted on holder and gold-sputtered in a Hitachi sputtering ion exchanger (HITACHI S-4800, Tokyo, Japan). The sensilla on the antennae were examined by scanning electron microscopy (SEM, FEI Quanta 200, quanta, Germany).

### Transmission electron microscope (TEM) observations

The antennae used for transmission electron microscope (TEM) were cut into a few pieces and immediately placed into the fixation solution [paraformaldehyde (4%) and glutaraldehyde (2.5%) in 0.1 M PBS pH 7.4] for 24 hr, then post-fixed with 1% OsO_4_ in 0.1 M PBS (pH7.4) at 4°C for 1 hr. The specimens were dehydrated in a graded series of ethanol (30%, 50%, 70%, 80%, 90%, 95%, 100%) for 10 min each, then soaked twice in acetone 10min for each and embedded in Epoxide 618 resin through mixtures of 2:1, 1:1, 1:2 of 100% acetone and Epoxide 618 resin, and kept in pure Epoxide 618 resin overnight. Polymerization was accomplished at 30°C for 6 hr; 50°C for 6 hr and at 60°C for 24 hr in tightly closed gelatine capsules. Ultrathin sections were cut with a diamond knife on a Leica EM UC6 microtome (Leica, Wetzlar, Germany) and mounted on Formvar-coated grids. The specimens were observed on a Hitachi H-7500 TEM (Hitachi, Tokyo, Japan).

### Data analysis

The number and width of different sensilla were made using Photoshop CS5 image processing software, and length of each antennal segment was measured using Image-Pro Plus (Ver.6.0) (Media Cybernetics, Rockville, USA) software, with the minimum value of scale is 0.01 mm and report error as ±0.005 mm. Pictures were only adjusted for brightness and contrast. The numbers of different sensilla on the antennae and the length of antennae segment between females and males were determined using independent Student's t-tests [25]. SPSS 19.0 (SPSS, Chicago, IL) was used to analysis the data.

## Results

### General morphology of antennae

**Fig 1:**
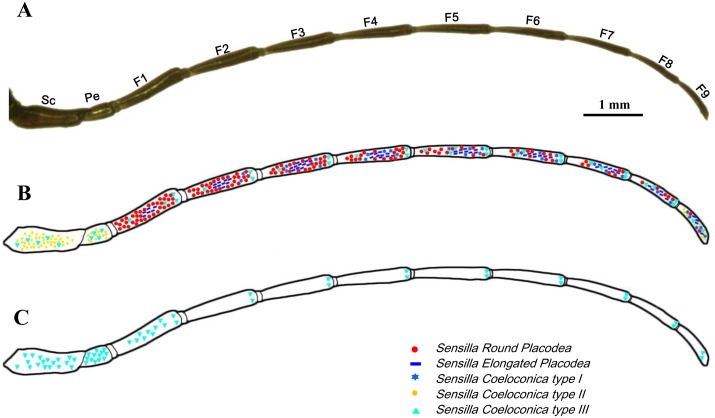
Overview of the general morphology and five types of sensilla distribution on the antenna of male *C*. *japonicus* Sharp. (A) External morphology of the male antenna under the light micrograph; the schematic distribution of various types of sensilla on the dorsal side (windward side) (B) and ventral side (lee side) (C). Sc: scape, Pe: pedicel, F1-F9: flagellum 1–9.

**Fig 2:**
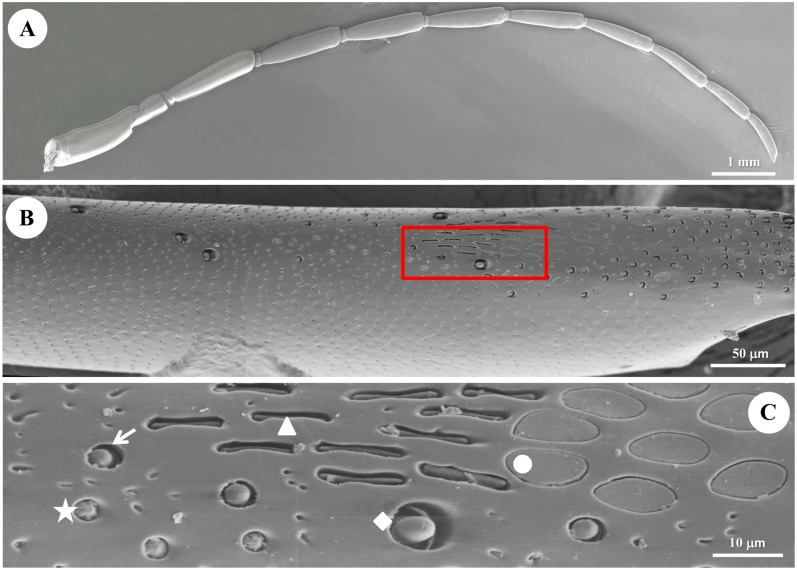
Scanning electron microscope (SEM) of general structure of the antenna in male diving beetle *C*. *japonicus* Sharp. (A) External morphology of the antenna under SEM; (B) Flagellum 9 of the antennae under SEM, and the area in the red rectangle is enlarged at higher magnification in (C), showing round s. placodea (round dot), elongated s. placodea (triangle), type I s. coeloconica (pentagram), type II s. coeloconica (arrow), and type III s. coeloconica (diamond).

**Fig 3:**
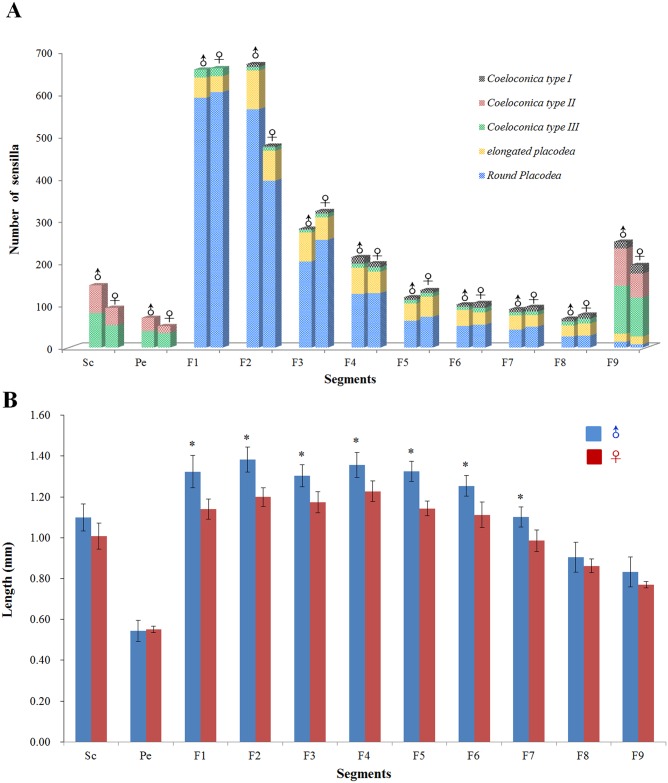
The sensilla distribution (A) and length of each segment (B) between males and females of diving beetle *C*. *japonicus* Sharp. Sc: scape, Pe: pedicel, F1-F9: flagellum 1–9. * indicates significant differences between sexes according to student’s t-test (P<0.05).

The antennae of diving beetle consist of a scape (the 1^st^ antennomere), a pedicel, and a flagellum. The flagellum is composed of 9 segments, named as F1 to F9 (Fig 1A). Five types of sensilla were identified on the antenna of *C*. *japonicus* with similar morphology and ultrastructure in both males and females (Figs 1 and 2). The distribution and morphological features of these five types of sensilla are reported in Figs 1B, 1C, 2 and 3A and Table 1. Besides sensilla, we also detected two clusters of bristles at the first two segments of the antenna. A cluster of Böhm’s bristles occurs on the scape while the other cluster of bristles is located at the basal part of the scape (S1 Fig). Sexual dimorphism occurs not only in the number of antennal sensilla (Table 1), but also in the length of the antennae (Fig 3B). In general, males have a larger number of each type of sensilla than in females except the type I s. coeloconica (*P*<0.05) (Table 1). The average length of the male antennae is significantly longer than that of the female antennae, especially in F1 to F7 (Fig 3B).

**Table 1:** The morphological feature and number of sensilla on the antennae of adult *C*.*japonicas* Sharp.

Type of sensilla (s)		s. placodea		s. coeloconica			Total sensilla	Proportion of s. placodea
		Round	Elongated	Type I	Type II	Type III		
Morphological feature (male)	Length (*μ*m)	---	11.26±1.05	---	---	---	---	---
	Width (diameter) (*μ*m)	7.52±0.88	1.11±0.44	3.08±0.33	3.18±0.25	5.96±0.46	---	---
	Shape of tip	Flat	Rindge	Blunt	Flat	Blunt	---	---
Number of sensilla	Male	1677.00±36.04a	427.00±10.44a	66.33±3.79a	185.00±4.36a	308.67±15.53a	2664.00±55.56a	78.98%
	Female	1589.67±17.89b	363.67±9.50b	77.67±2.89b	114.33±3.06b	264.33±1.53b	2409.67±27.97b	81.11%

Note: Data are presented as mean± S.D. (n = 10 for morphological feature and n = 3 for the number of sensilla); Data in the same columns (number of sensilla) followed by different letters are significantly different between sexes according to student’s *t*-test (*P*<0.05); --- indicates no data available.

### Structure of sensilla

Based on the morphological characteristics, the sensilla identified on the antennae of the adult diving beetle *C*. *japonicus* can be classified into five types: two types of s. placodea (round and elongated) and three types of s. coeloconica (type I-III).

**Fig 4:**
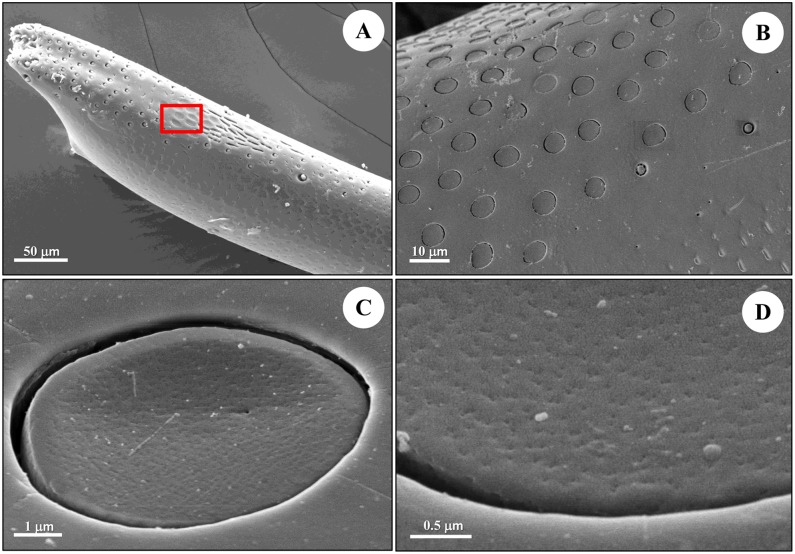
Scanning electron microscope (SEM) of round s. placodea in male diving beetle *C*. *japonicus* Sharp. (A) Flagellum 9 of the antennae under SEM showing round s. placodea in red rectangle. (B, C) Close-up view of the round s. placodea on the dorsal side of antennal flagellum. (D) Pores on the surface of the round s. placodea, with density of 60 pores/μm^2^.

**Fig 5:**
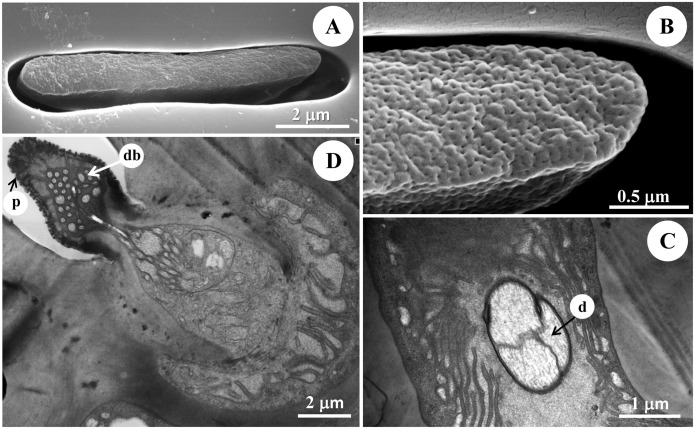
The morphology and ultrastructure of the elongated s. placodea of diving beetle *C*. *japonicus* Sharp. (A) SEM of the elongated s. placodea, showing many pores on the surface that perforate the outer cuticle. (B) The multiple pores under the SEM with density of 170 pores/μm^2^. (C) Longitudinal section through the elongated s. placodea, showing three dendrites enclosed in the dendrite sheath. (D) Ultrastructure of the enlongated s. placodae under TEM showing the intercuticular space between the inner and outer cuticles, where the dendrites lose their arrangement, with the dendritic branches occupy the whole space, and the cuticular wall of these sensilla is around 0.5 μm. p: pore, d: dendrite, db: dendritic branches.

**Fig 6:**
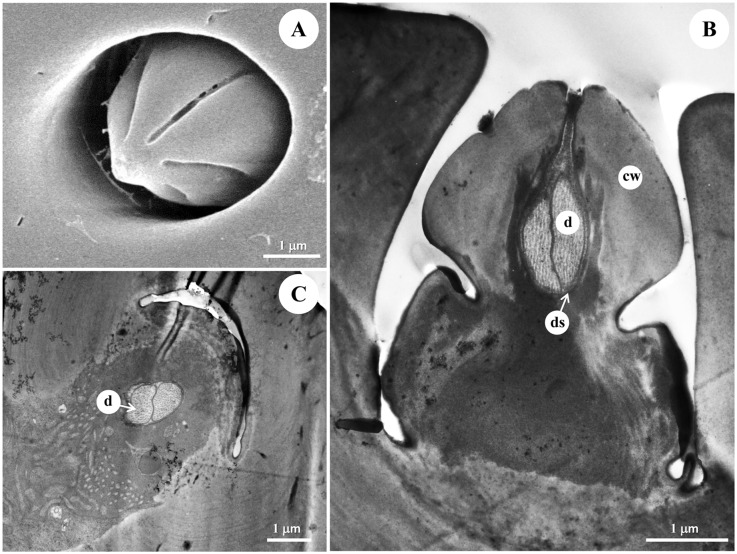
The morphology and ultrastructure of the type I s. coeloconica. (A) SEM of the type I s. coeloconica of male diving beetle *C*. *japonicus* Sharp, which is characterized as a flame-like peg, sitting inside a chamber. Longitudinal (B) and cross section (C) of the sensilla under TEM showing the thick cuticular wall and two sensory neurons, tightly embedded by a thick dendrite sheath, are presented inside each peg. Cw: cuticle wall, ds: dendritic sheath, d: dendrite.

**Fig 7:**
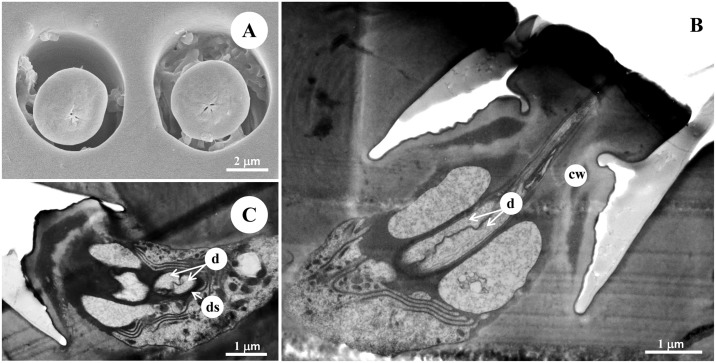
The morphology and ultrastructure of the type II s. coeloconica of male diving beetle *C*. *japonicus* Sharp. (A) SEM of the type II s. coeloconica, containing a peg with flower-slit on the flat surface. Longitudinal (B) and cross section (C) of the sensilla under TEM showing it contains two bipolar neurons. According to (B), both dendrites reach the tip of the sensillum peg. The cross section in (C) showed two sensory dendrites are tightly embedded by the dendrite sheath. cw: cuticle wall, d: dendrite, ds: dendrite sheath.

**Fig 8:**
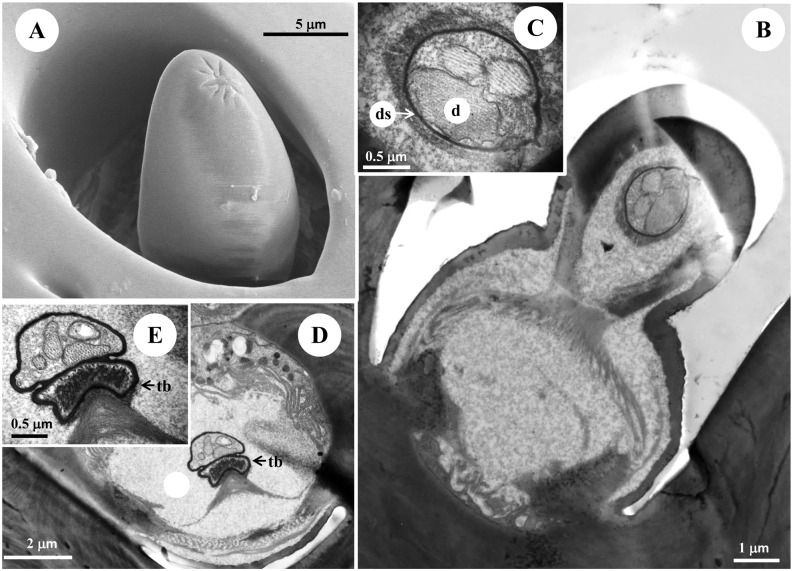
The morphology and ultrastructure of the type III s. coeloconica of male diving beetle *C*. *japonicus* Sharp. (A) SEM of the type III s. coeloconica showing this type of sensillum is characterized externally by a smooth peg, with about 10μm in height. (B) Four dendrites are located in two separated cavities, and three of them reach the tip of the peg, which was shown at higher magnification in (C), while one of them ends in a tubular body beneath the base of the peg (D), shown at higher magnification in (E). d: dendrite, tb: tubular body, ds: dendritic sheath.

Both round s. placodea and elongated s. placodea are equipped with numerous pores on the sensilla surface (Table 1). But they are different in their morphological shape: the round s. placodea is elliptical plate-shaped (Figs 2C and 4), while the elongated s. placodea is sausage-shaped (Figs 2C and 5A). The s. coeloconica is characterized as a peg-in-pit sensillum, where the peg is encircled by a socket. Three types of s. coeloconica have different types of pegs (Figs 6A, 7A and 8A).

S. placodea and the type I s. coeloconica are detected along all flagellar segments, except that the first segment lacks the type I s. coeloconica (Fig 1B). The dorsal surface of the antenna is generally occupied by a large number of both types of s. placodea, mixed with each other along all nine flagellar segments. In particular, they are highly concentrated on the 1^st^-3^rd^ flagellar segments in both male and female antennae (Fig 1B). In contrary, the type II s. coeloconica are found only on the dorsal side of the scape, pedicel, and F9 segments with low abundance. The type III s. coeloconica can be found on each segment of the whole antenna (Figs 1B, 1C and 3A), and they are the only type of sensillum located on the ventral side of the antenna (Fig 1C).

### Round s. placodea

**Fig 9:**
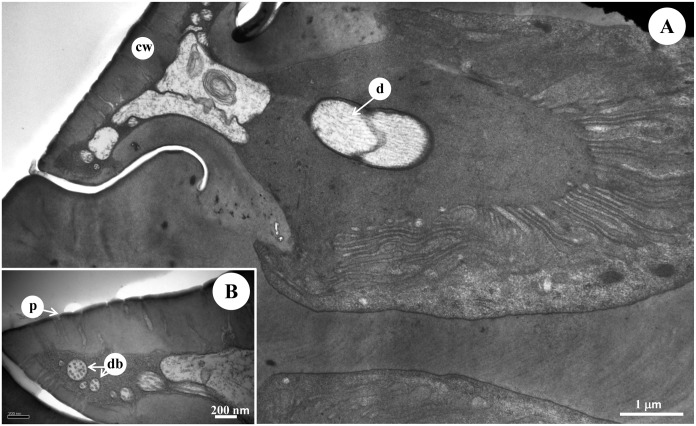
Longitudinal section of the round s. placodea of female diving beetle *C*. *japonicus* Sharp under transmission electron microscope (TEM). The dendrites are clustered into a group, containing 2 bipolar neurons (A). Numerous pores on the surface of sensilla that perforate the outer cuticle (B). p: pore, d: dendrite, db: dendritic branches, cw: cuticle wall.

The round s. placodea are flat circular plates surrounded by a deep ditch (Fig 4), with the width ranging from 7.52±0.88 μm in diameter (Table 1). They are located on the dorsal side of all 9 flagellar segments and are the most abundant antennal sensilla in *C*. *japonicus*, with 1677.00±36.04 in males, and 1589.67±17.89 in females (Fig 3A, Table 1). The density on the first two proximal flagellar segments is extremely high, around 63–68% of total number, gradually decreasing toward the distal end of the flagellum (Figs 1B and 3A). These sensilla present a relatively thick plate, ranging from 0.5 μm to 1.0 μm. Many pores are located on their surface, with a density of approximately 60 pores/μm^2^ (Fig 4D). The pore channels penetrate the thick cuticle, extend into the sensillum, and eventually reach out towards the dendritic branches (Fig 9A). Two dendrites of bipolar neurons within the placoid sensillum, clustered and enclosed by the dendrite sheath (Fig 9A), reach the area beneath the sensilla cuticle, where their distal ends become separated. The number of microtubules inside the dendritic branches ranges from several to a dozen (Fig 9B).

### Elongated s. placodea

These sensilla are cuticular ridges slightly elevated above the antennal surface, and surrounded by a grooved gap (Figs 2B, 2C and 5). They appear as narrow ridges with 11.26±1.05 μm in length and 1.11±0.44 μm in width, and aligned generally in parallel with the antennal axis (Table 1, Figs 2B and 5A). Elongated s. placodea are less numerous on the antennae compared to the round s. placoidea (Fig 3A, Table 1), and always located together with round s. placodea along the F1-F9 segments (Fig 1B). The cuticular wall of these sensilla is around 0.5 μm, with numerous pores which partially penetrate the outer cuticle, approximately 170 pores/μm^2^ (Fig 5A and 5B). Three morphologically similar dendrites are clustered into a group (Fig 5C). When the dendrites enter the median channel, they branch and turn toward the distal end of the sensillum, occupying the whole space (Fig 6D). The space beneath the pore is fill with sensillum lymph where the dendritic branches located.

### Type I s. coeloconica

Type I s. coeloconica are found along the F2-F9 segments, with the numbers 66.33±3.79 in male antennae and 77.67±2.89 in female antennae (Table 1), with an intensive distribution on the tip of F9 segment in both sexes (average 18 in males, 22 in females) (Fig 3A). These are typical peg-in-pit sensilla, characterized externally by a round aperture with a diameter of 3.08±0.33 μm (Table 1). The peg is flame-like in shape, with a diameter of 2.7–3.4 μm, inserted 4–5 μm deep into the chamber (Fig 6A). Two sensory neurons, tightly embedded by a thick dendrite sheath, almost fill the whole lumen, without leaving any space for sensillar liquor (Fig 6B), while the gap between them is filled by dense material (Fig 6C).

### Type II s. coeloconica

Type II s. coeloconica are scattered only on the two proximal segments (scape and pedicel) and the F9 segment (Table 1, Figs 1B and 3A). These are also peg-in-pit sensilla, presenting a peg with flower-slit on the flat surface, externally different from the type I s. coeleconica (Fig 7A). The type II s. coeloconica contain two bipolar neurons at the basal region, and both dendrites reach the tip of the sensillum peg (Fig 7B). These two sensory dendrites are tightly embedded inside the dendrite sheath, with no space left for sensillum lymph (Fig 7B and 7C).

### Type III s. coeloconica

These sensilla are scattered along all segments of the entire antennae (Figs 1B, 1C and 3A), with the numbers about 308.67±15.53 in male antennae and 264.33±1.53 in female antennae (Table 1). Type III s. coeloconica are characterized externally by a smooth peg, ending with a round tip (Fig 8A). The peg is about 10 μm in height and surrounded by a deep chamber, with only the top (around 2 um) protruding outside the cuticle surface (Fig 8A and 8B). They are innervated by 4 receptor cells, three of which are embedded inside the dendrite sheath, and extend to the distal end of the peg (Fig 8B and 8C). The dendrite of the fourth receptor cell terminates in a tubular body at the base of the peg in a separated dendritic sheath (Fig 8D and 8E).

## Discussion

In this study, we examined the morphology and ultrastructure of antennal sensilla of the diving beetle *C*. *japonicus* Sharp. Five types of surface sensilla, including two types of sensilla placodea and three types of sensilla coeloconica, were identified and characterized in details by scanning electron microscope (SEM) and transmission electron microscope (TEM). Males bear a longer antenna with more sensilla located comparing to the females. Besides this, the size, shape, and distribution pattern of sensilla on the antennae are similar between males and females.

The structure characteristics of sensilla placodea vary greatly among different insect groups, however, all sensilla placodea share a common feature- a large number of pores on the sensilla surface [26, 27]. Multiporous (MP) sensilla were also found on the antenna of diving beetle *G*. *occidentalis*, where a cluster of MP sensilla were sunk in the sockets and flattened to a plate like surface externally [22]. They were classified into 2 types: multiporous pitted (MPp) sensilla equipped with a pitted external surface, and multiporous grooved (MPg) sensilla having longitudinal surface grooves, which are similar to the round and elongated sensilla placodea that we identified in the *C*. *japonicus* Sharp, respectively. In addition, the MP sensilla were also described in diving beetle, *A*. *sulcatus* [23, 28] and *D*. *marginalis* [29]. In general, the MP sensilla were considered to sever as chemoreceptors during the chemical communication [12]. It was documented that the sensilla placodea on the antennae of *D*. *marginalis* responded to a variety of chemicals [16, 29]. Being excited by females produced pheromone, male *D*. *marginalis* would quickly move their antennae and palpi and increase their swimming speed toward to the female [30]. In another study, the electrophysiological recordings on a terrestrial beetle *Anomala cuprea* indicates that the species specific pheromone was detected by sensilla placodea in the male antennae [31].

In the diving beetle *C*. *japonicus* Sharp, male antenna are equipped with significantly more sensilla placodea than female antenna (Table 1), which may suggest potential functions of these types of sensilla in chemical communication during its precopulatory and copulatory activities [15]. Interspecific chemical interactions are generally mediated by allelochemicals, such as allomone and kairomone. Predaceous diving beetles, including *C*. *japonicus* Sharp mainly rely on these chemical clues to detect, attack, capture, and ingest their prey, which was proved to be functional even in total darkness [15, 32]. However, the chemical stimuli to which these sensilla placodea respond are still unknown. Further studies of immunocytochemistry and molecular biology are necessary to clarify the function of sensilla placodea in intraspecies and interspecies chemical communication in the diving beetle *C*. *japonicus* Sharp.

Three types of coeloconica sensilla were identified on the antennae of the diving beetle *C*. *japonicas*. The type I s. coeloconica are scattered along the 2^nd^-9^th^ flagellar segments, bearing a flame-like peg, while those of type II s. coeloconica are predominantly scattered on the scape, pedicel, and the F9 segment. These types of sensilla were also reported on the surfaces of maxillary palpi of diving beetle *C*. *fimbriolatus Say*, where they present a similiar peg, surrounded by a deep and thick circular furrow, coupled with a wall around, and located near the tip of palpi [33]. The peg of types I and II s. coeloconica are innervated by two neurons, packaged tightly within the thick cuticle, a typical feature of mechano-sensitive receptors [34, 35], indicating the possible role associates with touch-reception that these types of sensilla may play. A similar sensillum, named F peg, well described on the antennae of the bed bug, *Cimes lectularius* L, is tightly covered by cuticle, with 2–3 cells enclosed [36]. Thermoreceptors generally have a triad of neurons, ending at the base and forming lamellar structures. However, in our species only two dendrites were identified. As the adults *C*. *japonicas* are generally good fliers and can disperse across great distances to find new water habitats [37, 38]. During their dispersal flight, temperature is a major determinant of patterns of distribution for dytiscid species [39]. Therefore, we cannot exclude the potential function of these two dendrites as thermoreceptors as well. As the scape and pedicel are located at the joint between the antenna and the head (Fig 1), the specific location of the type II s. coeloconica on these structures might indicate a function in detecting general stresses in the cuticle produced by the movement of the antennae, which is similar to the function of Bӧhm’s bristles (S1 Fig) that may play [40].

The aporous type III s. coeloconica are distributed on both dorsal and ventral sides of the entire antennae, and show no distinct differences in location between the sexes (Fig 1). Morphologically similar sensilla were also found on the surfaces of maxillary palpi of the same species (S2 Fig) and of *C*. *fimbriolatus Say* [33]. The fine structure of the Type III s. coeloconica shows that four dendrites are located in two separated cavities, and one of them ends beneath the basement of the peg, while the other three reach the tip of the peg (Fig 8). The similar sensilla were detected on the distal antennal segment of the diving beetle *G*. *occidentalis* [21]. Based on the aporous characteristics and previous publications, the type III s. coeloconica in *C*. *japonicas* may have both mechano-receptive and gustatory functions [24, 41, 42]. Further studies of electrophysiology and behaviour are required to confirm the functions of antennal sensilla in this species.

## Supporting information

S1 Fig The morphology of two clusters bristles of diving beetle *C*. *japonicus* Sharp. (A) SEM for the scape and scape segments with one group of the bristles located at the basal part of the scape, shown in the solid red rectangle which is enlarged at higher magnification in (B), and the other cluster of Böhm’s bristles occurring on the scape shown in the dashed red rectangle, which are enlarged at higher magnification in (C) and (D). Sc: scape, Pe: pedicel.
(TIF)

S2 Fig The morphology of maxillary palpi of diving beetle *C*. *japonicus* Sharp. (A) The tip of maxillary palpi under SEM showing the uneven surface, where numerous sensilla are located. The sensillum in the red square was enlarged at higher magnification in (B), with similar characters as the type III s. coeloconica on the antennae.
(TIF)
