# Supplementary figures and images for: Ultrastructure and morphology of antennal sensilla of the adult diving beetle Cybister japonicus Sharp
Source: PLoS One. 2017 Mar 30;12(3):e0174643. doi: 10.1371/journal.pone.0174643 (PMC5373609; doi:10.1371/journal.pone.0174643)

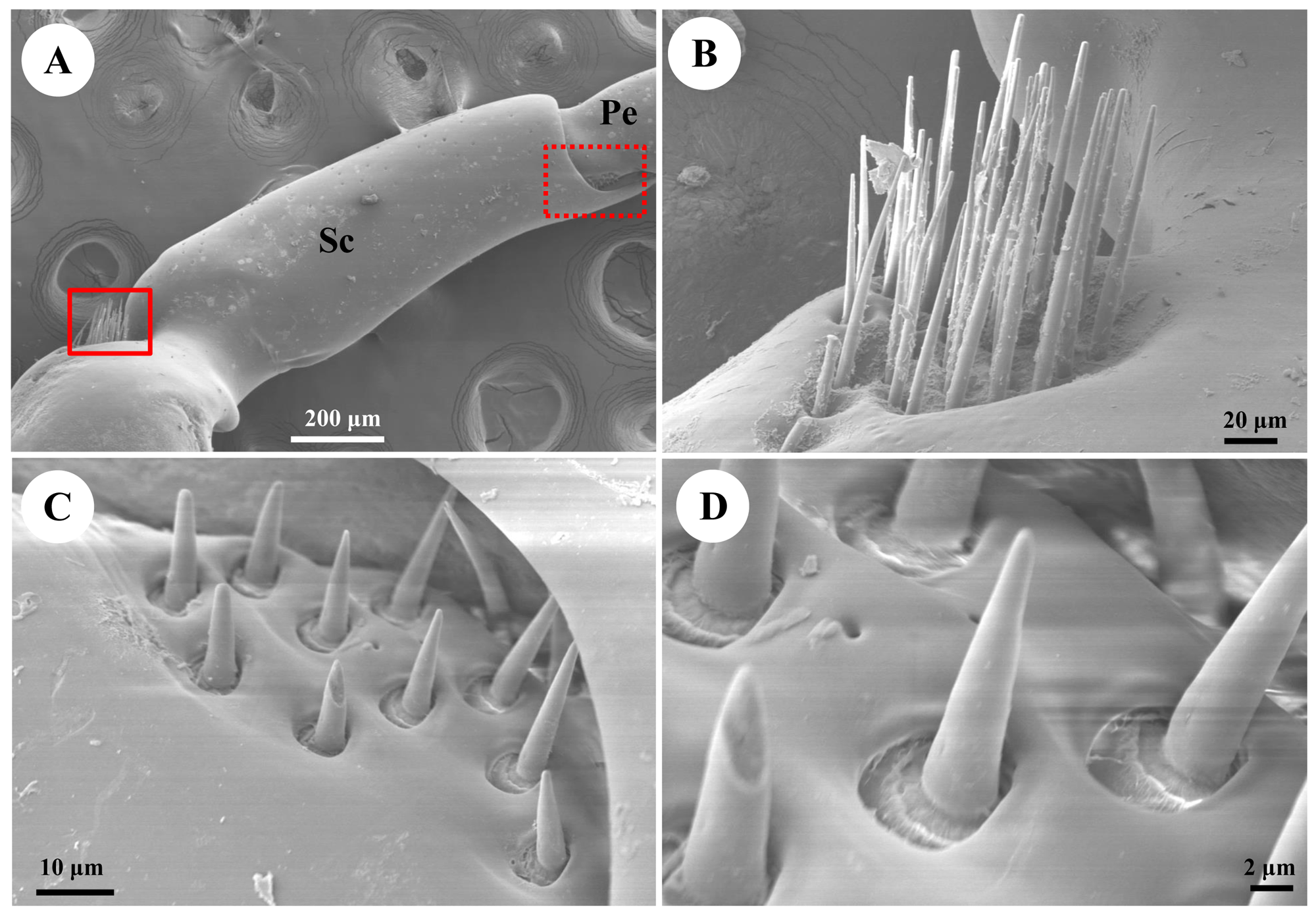

Supplement: S1 Fig — (A) SEM for the scape and scape segments with one group of the bristles located at the basal part of the scape, shown in the solid red rectangle which is enlarged at higher magnification in (B), and the other cluster of Böhm’s bristles occurring on the scape shown in the dashed red rectangle, which are enlarged at higher magnification in (C) and (D). Sc: scape, Pe: pedicel. (TIF) [file pone.0174643.s001.tif]

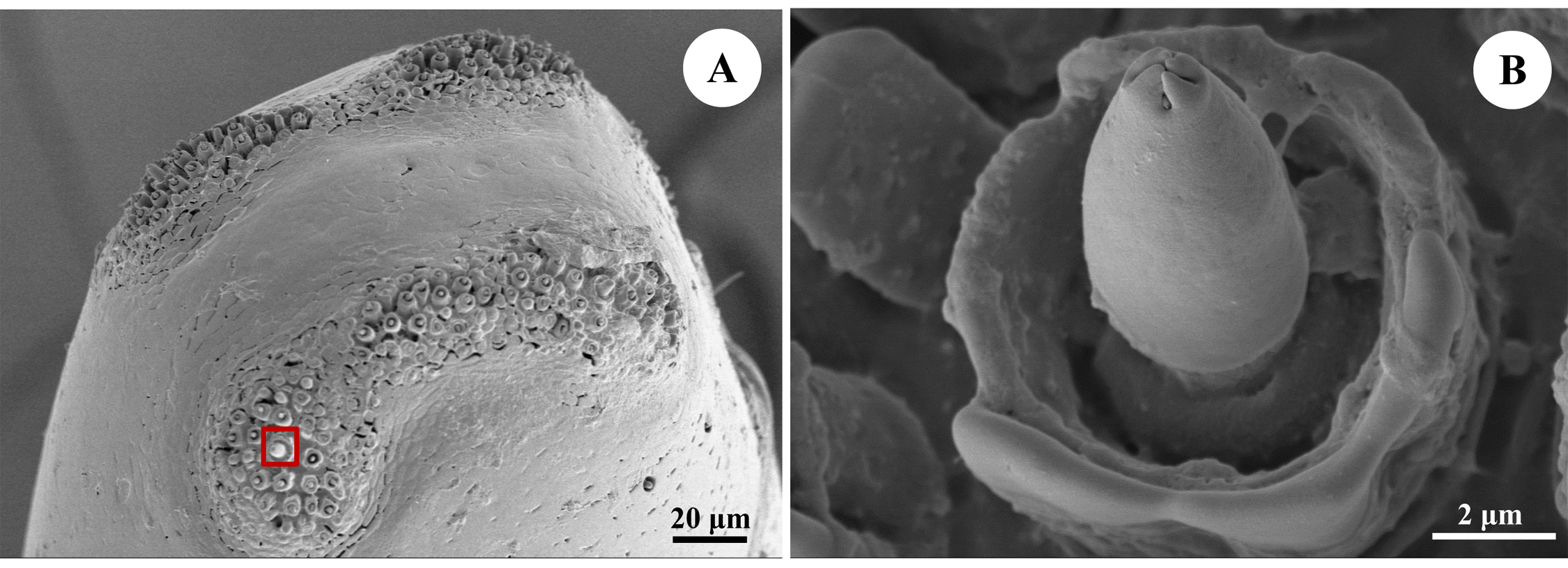

Supplement: S2 Fig — (A) The tip of maxillary palpi under SEM showing the uneven surface, where numerous sensilla are located. The sensillum in the red square was enlarged at higher magnification in (B), with similar characters as the type III s. coeloconica on the antennae. (TIF) [file pone.0174643.s002.tif]
